# Robust 3D lane detection in complex traffic scenes using Att-Gen-LaneNet

**DOI:** 10.1038/s41598-022-15353-w

**Published:** 2022-06-30

**Authors:** Yanshu Jiang, Qingbo Dong, Liwei Deng

**Affiliations:** Heilongjiang Provincial Key Laboratory of Complex Intelligent System and Integration, School of Automation, Harbin University of Science and Technology, Harbin, 150080 China

**Keywords:** Engineering, Electrical and electronic engineering

## Abstract

Robust 3D lane detection is the key to advanced autonomous driving technologies. However, complex traffic scenes such as bad weather and variable terrain are the main factors affecting the robustness of lane detection algorithms. In this paper, a generalized two-stage network called Att-Gen-LaneNet was proposed to achieve robust 3D lane detection in complex traffic scenes. The Efficient Channel Attention (ECA) module and the Convolutional Block Attention Module (CBAM) were combined in this network. In the first stage of the network, we improved the semantic segmentation network ENet and proposed the weighted cross-entropy loss function to solve the problem of ambiguous distant lane segmentation. This method improved Pixel Accuracy to 99.7% and MIoU to 89.5%. In the second stage of the network, we introduced the interpolation loss function to achieve accurate lane fitting. This method outperformed existing detection methods by 6% in F-score and Average Precision on the Apollo Synthetic dataset. The proposed method achieved better overall performance in 3D lane detection and was applicable to broader and more complex traffic scenes.

## Introduction

In recent years, with the rise of autonomous driving technology, the transportation industry is developing rapidly in the direction of intelligence and autonomy. One of the prerequisites for these directions is the automatic detection and identification of various elements in the traffic scenes. Lanes are essential traffic signs, so accomplishing robust detection of lanes in complex traffic scenes is the key to implementing advanced autonomous driving technologies^1–3^.

Most of the current lane detection methods only stay at the 2D level, and the emergence of 2D lane detection datasets such as Tusimple^4^ and Culane^5^ make this research direction develop more rapidly. Researchers were committed to improving the detection accuracy of lane, and attention modules such as Dual Attention and SAD^6–10^ were added to semantic segmentation networks. These methods used spatial or channel correlation to assist in accurate lane fitting. 2D lane detection algorithms usually perform semantic segmentation of the image first^5,11–13^ and then convert the driver's view image into the bird's eye view using the inverse perspective transformation^14,15^. Curve fitting uses polynomials in the bird's eye view^16,17^ and the output detection results are approximate curves of the 3D lane in the real scenes. Real-time performance is an essential metric for evaluating lane detection algorithms. The CondLaneNet proposed by Liu et al.^18^ achieved a detection rate of 220 FPS on the Culane dataset, and the PolyLaneNet proposed by Lucas Tabelini et al.^19^ achieved a detection rate of 115 FPS on the Tusimple dataset. These lane detection algorithms used many assumptions on lane properties such as flat roads and uniform lighting. Due to the above assumptions, the existing lane detection technologies have poor robustness and provide false perceptions when the vehicle is driving up and down hills, curve lanes, and complex traffic scenes such as rain or snow, in general lack adaptive capabilities compared to drivers.

**Figure 1:**
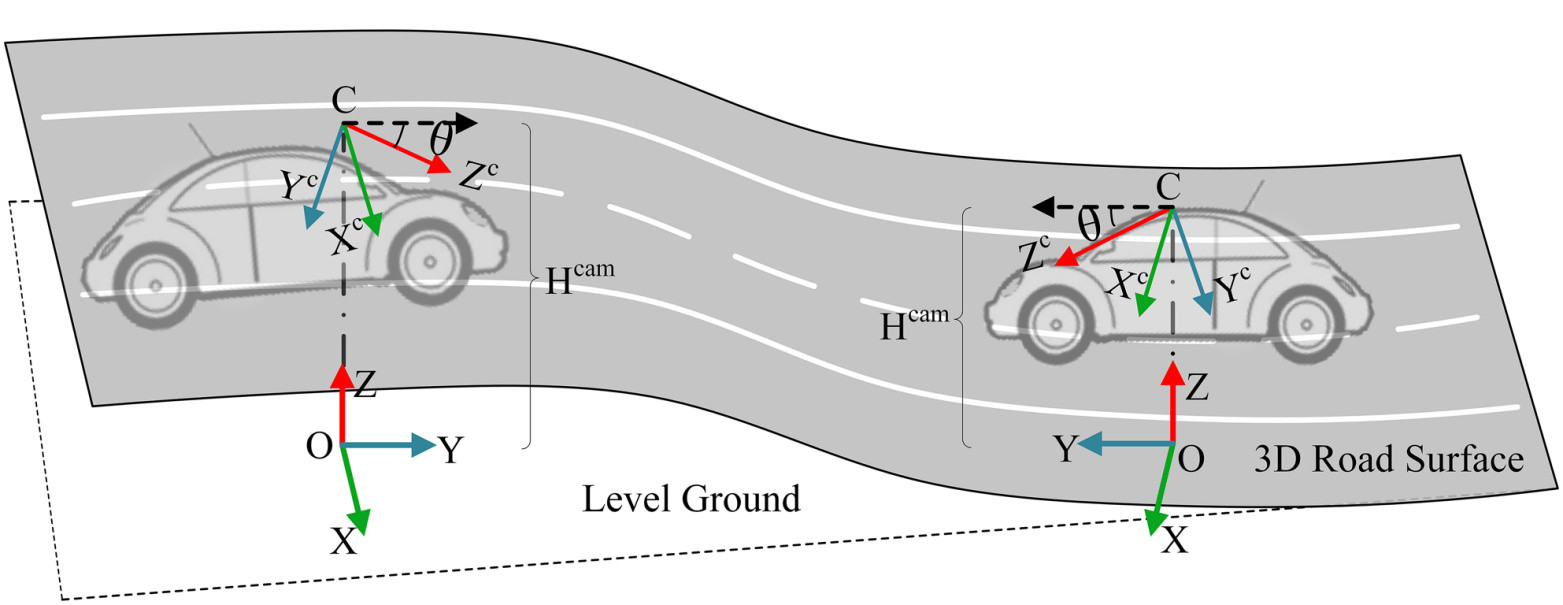
In-vehicle camera position and vehicle coordinate frame. This figure was drawn by author Qingbo Dong.

In recent years, scholars have started to research 3D lane detection, which mostly relies on the geometric relationship between the in-vehicle camera settings and the road surface, as shown in Fig. 1. 3D-LaneNet^20^ was one of the first end-to-end network frameworks proposed in this research direction, which implemented IPM transformation internally and introduced the concept of anchor representation. 3D-LaneNet implemented end-to-end training with image views and bird's eye views in parallel, and achieved superior results in complex traffic scenes such as lane merging and splitting. Netalee Efrat et al.^21^ proposed a camera-based DNN method. This method followed the parallel structure in 3D-LaneNet and decomposed the lanes into the lane line segments using grids in the bird's eye view. In this approach, adjacent grids will have overlapping perceptual fields, so the lane line segments of adjacent grids can be clustered into complete lanes. The two-stage network proposed by Guo et al.^22^ computed 3D lane point coordinates using the geometric transformation between the in-vehicle camera coordinate system and the vehicle coordinate system. This method was beginning to be applied to unseen scenes.

Although existing lane detection algorithms have been extended to the 3D level, there are still many problems. When segmenting lane images, we will face the problem of ambiguous distant lane segmentation due to complex traffic scenes such as hills and curve lanes. In addition, the great difference in the number of lane and background pixels lead to unclear lane edge segmentation. When predicting 3D lane structure, the model has poor generalization ability in unseen scenes and complex weather. Therefore, we designed a two-stage network that focused on segmentation of 3D lane and 3D lane coordinates prediction. In the first stage, we improved the lightweight semantic segmentation network ENet^23^ and introduced the ECA attention module^24^ in the decoder part of ENet to improve the segmentation effect by enhancing the lane and background discrimination ability of the model. The CBAM attention module^25^ was introduced between the top view encoding layer and the prediction head of the second stage geometric encoding subnetwork to aggregate more global information to enhance the model’s generalization ability. We designed the weighted cross-entropy loss function to constrain the problem of the unbalanced number of lane and background pixels in the semantic segmentation process. We also introduced the interpolation loss function^26^ to solve the problem of poor local fit of the lanes.

The contributions of this paper are summarized as follows:

- A two-stage 3D lane detection network was designed with superior generalization performance of the model for a wider range of traffic scenes.
- The ECA attention mechanism and the CBAM attention mechanism were introduced in the two stages, which improved the segmentation effect and prediction accuracy of the network accordingly.
- The weighted cross-entropy loss function and the interpolation loss function were improved in the two stages to enhance the model’s generalization ability.

The main goal of this research is to design a 3D lane detection algorithm with more robust performance to provide a model for more advanced autonomous driving techniques. In this paper, the second section describes the network architecture and the main methodology, the third section shows the experimental data and result plots, and last section illustrates our conclusions.

## Methods

### Attention mechanism

#### ECA attention module

**Figure 2:**
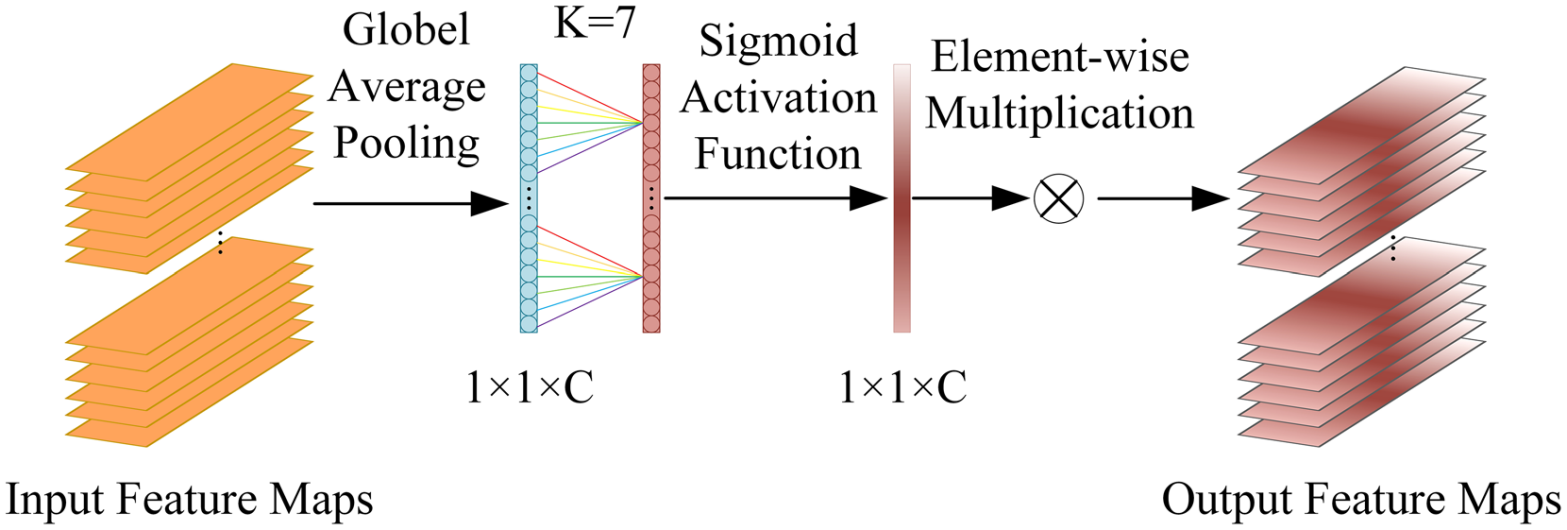
The structure of the ECA module.

In the first stage of the network, we introduce the ECA attention module^24^ to assist the 3D lane segmentation. The structure of the ECA attention module is shown in Fig. 2. The overall structure after adding the ECA attention module is shown in Fig. 6. This module contains only nine parameters, which not only learns the correlation between different channels of the feature maps and improves the sensitivity of the network to the 3D lane structures. The ECA attention module first performs global average pooling of the input feature and then performs a one-dimensional convolution operation with a convolution kernel of $k$. The generated feature maps through the sigmoid activation function to obtain the weights of each channel, and the weights are multiplied with the corresponding elements of the input feature maps to obtain the output feature maps. The value of $k$ is determined adaptively by the channel dimension in the input feature maps, and the equation is as follows.

1 $$k = \psi (C) = \left| {\frac{{\log_{2} (C)}}{\gamma } + \frac{b}{\gamma }} \right|_{odd}$$

where $\left| n \right|_{odd}$ indicates the nearest odd number $n$. The parameters of $\gamma$ and $b$ are set to 3 and 2, and $C$ is the number of channel dimensions.

#### CBAM attention module

**Figure 3:**
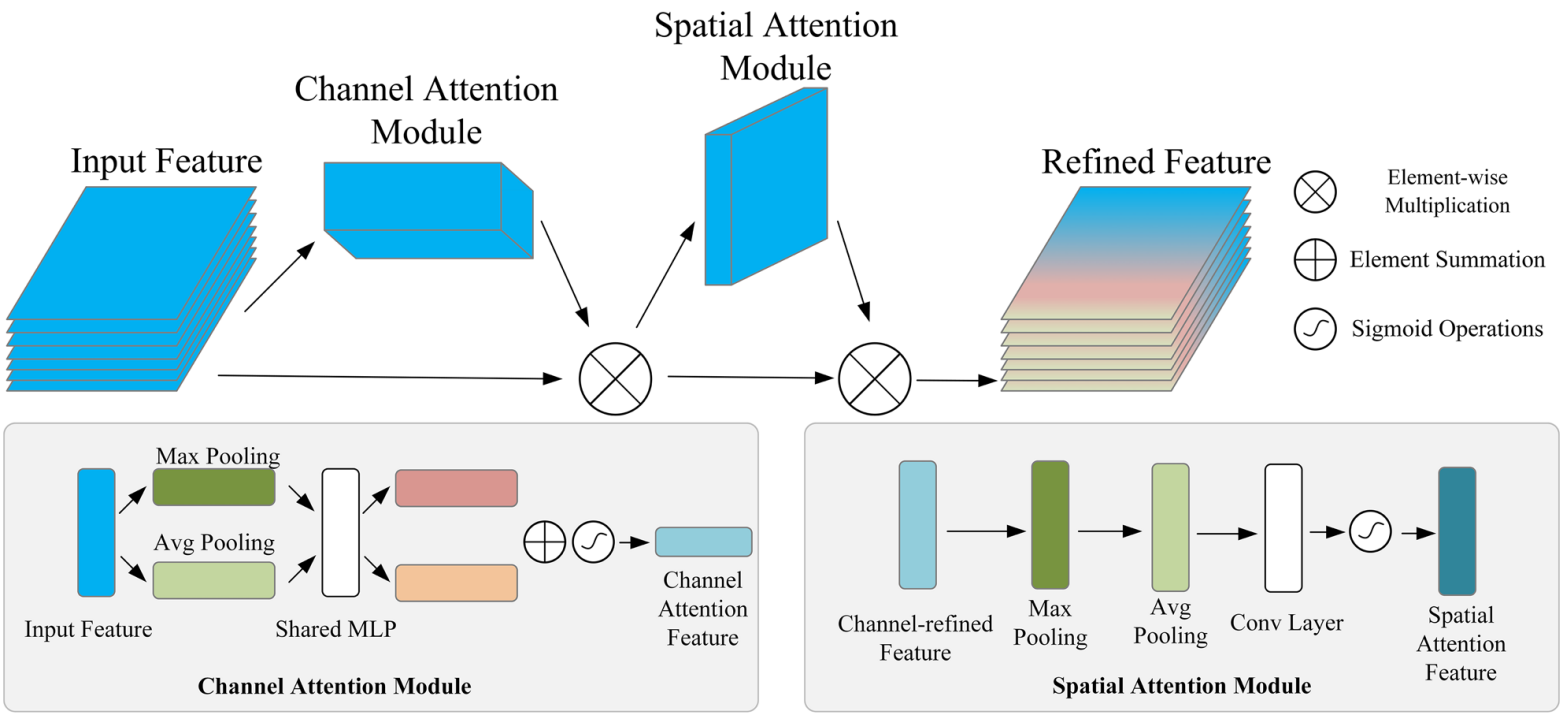
The structure of the CBAM attention module.

In the second stage of the network, we introduce the CBAM attention module^25^ to assist the 3D lane prediction. We add it between the Top-view Segmentation encoder and the lane prediction head. The overall structure after adding the CBAM attention module is shown in Fig. 7. The CBAM attention module consists of a spatial attention module and a channel attention module in series. The overall structure of the CBAM attention module is shown in Fig. 3. The outputs of the convolutional layer will first pass through the channel attention module to get the weighted results and then will pass through the spatial attention module to get the final weighted results. The CBAM attention module extracts more global information in both channel and spatial dimensions to predict 3D lane structures better.

The channel attention module performs the input feature’s global max pooling and global average pooling to obtain two one-dimensional vectors. These two vectors will pass through the shared multi-layer perceptron (MLP) and be summed, and finally the channel attention feature maps are generated using the sigmoid activation function. The channel attention feature maps are element-wise multiplied with the input feature maps to generate the input feature maps of the spatial attention module. In the spatial attention module, we first perform global max pooling and global average pooling operations on the channel dimension and concatenate the two results. The generated results are reduced to one channel through a convolutional layer and then through the sigmoid activation function to generate the spatial attention feature maps. The spatial attention feature maps are multiplied with the channel-refined feature maps to obtain the final refined feature maps. The following equations describe the channel attention module and the spatial attention module.

2 $$\begin{gathered} M_{c} (F) = \sigma (MLP(AvgPool(F)) + MLP(MaxPool(F))) \hfill \\ \, \quad \quad = \sigma (W_{1} (W_{0} (F_{avg}^{c} )) + W_{1} (W_{0} (F_{\max }^{c} ))) \hfill \\ \end{gathered}$$

where *σ* denotes the sigmoid activation function, $W_{0}$ and $W_{1}$ denote the weight matrix in the $MLP$, and $F_{avg}^{c}$ and $F_{\max }^{c}$ denote the average pooling feature and max pooling feature in the channel attention module.

3 $$\begin{gathered} M_{s} (F) = \sigma (f^{7*7} ([AvgPool(F);MaxPool(F)])) \hfill \\ \,\quad \quad = \sigma (f^{7*7} ([F_{avg}^{s} ;F_{\max }^{s} ])) \hfill \\ \end{gathered}$$

where $f^{7*7}$ denotes the convolution operation with a filter size of $7 \times 7$, $F_{avg}^{s}$ and $F_{\max }^{s}$ denote the average pooling feature and the max pooling feature in the spatial attention module.

### Geometric transformation and anchor representation

In this paper, the 3D lane is represented in the vehicle's coordinate system consisting of $X,Y,Z$ axes and the origin $O$. We use the height $H^{cam}$ and the pitch angle $\theta$ to indicate the camera's pose. The camera coordinate system is represented by $X^{c} ,Y^{c} ,Z^{c}$ and the origin $C$. $Z$ denotes the real height of a 3D lane. The 3D lane can be projected onto the image plane by projection transformation and then the lane image can be projected onto a flat road surface by planer homography to generate a bird's eye view. Due to the camera parameters are involved, the lanes in the bird's eye view have different $X,Y$ values compared to the 3D lanes in the vehicle's coordinate system. We set the bird's eye view as a special coordinate system defined by the $x,y,Z$ axes and the origin $O$. The geometric transformation between the coordinate system of the bird's eye view and the vehicle's coordinate system can be expressed by the following equation:

4 $$\left[ {\begin{array}{*{20}c} x \\ y \\ \end{array} } \right] = \left[ {\begin{array}{*{20}c} {\frac{{H^{cam} }}{{H^{cam} - Z}}} & 0 & 0 \\ 0 & {\frac{{H^{cam} }}{{H^{cam} - Z}}} & 0 \\ \end{array} } \right]\left[ {\begin{array}{*{20}c} X \\ Y \\ Z \\ \end{array} } \right]$$

**Figure 4:**
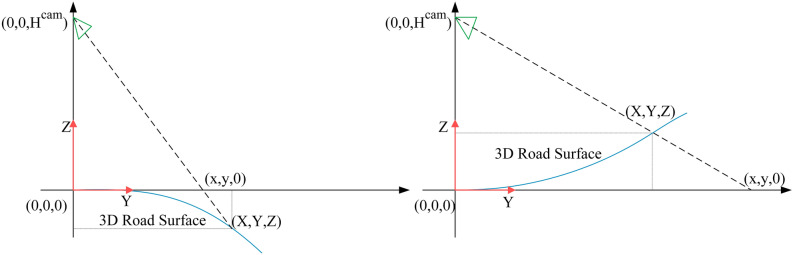
Geometric transformation.

The 3D lane coordinates are represented as the $x,y$ in the bird's eye view coordinate system and the real height $Z$. Since the geometric transformation is independent of the camera parameters, the geometric transformation holds whether the vehicle is driving on the uphill or downhill scenes. Using this geometric transformation, the lane coordinates in the bird's eye view can be mapped back to the real road coordinates. The geometric transformation is shown in Fig. 4.

**Figure 5:**
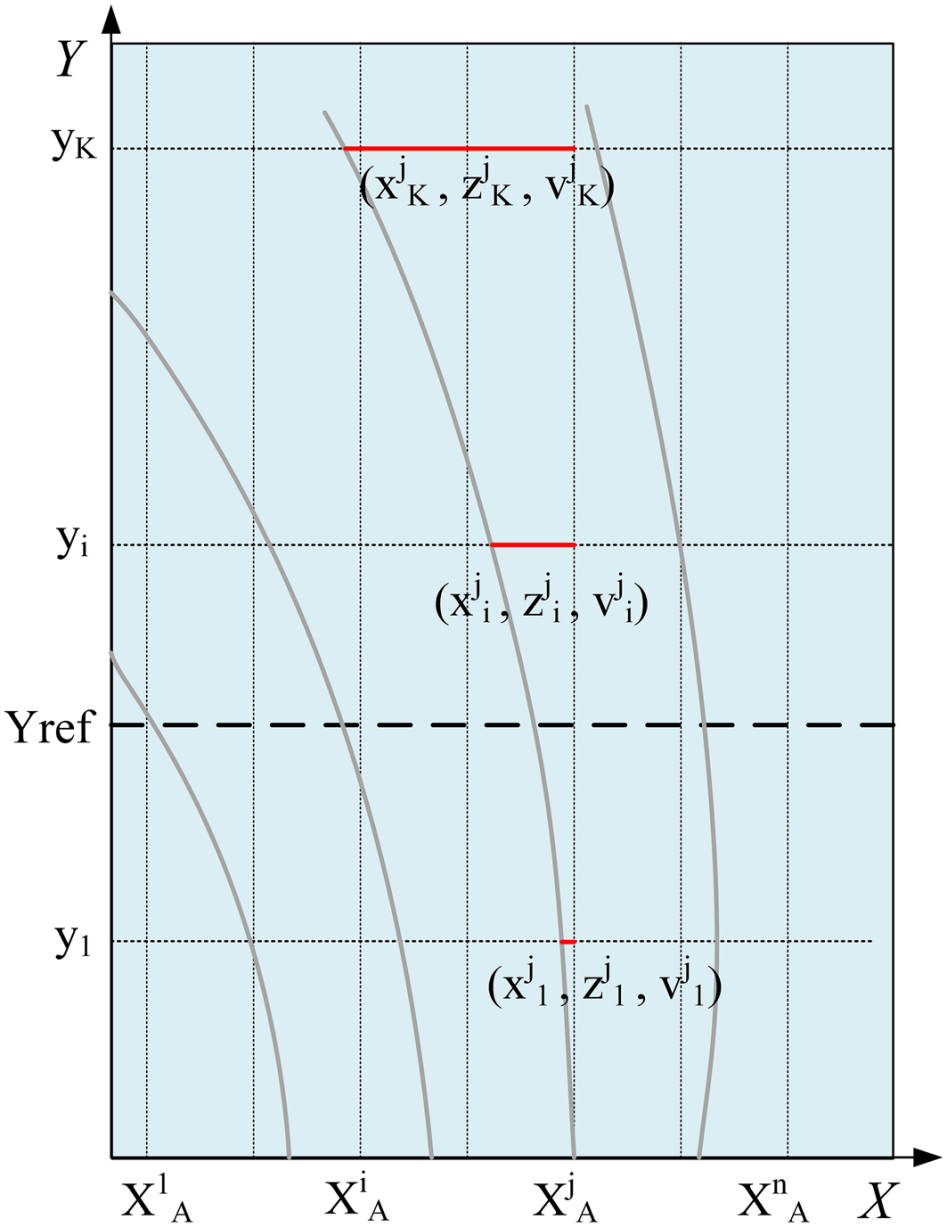
Anchor representation.

We use the anchor representation^22^ combined with the geometric transformation to compute real 3D lane coordinates, enabling our method to predict 3D lane structures in unseen scenes. The anchor representation as shown in Fig. 5. In this method, we predefine $n$ equidistant vertical lines on the x-axis to determine the position of the anchors and define $k$ fixed $y$ positions. When the predicted lane crosses the $Y_{ref}$ location, the ground-truth lane is associated with the nearest anchor based on the $x$ value. An anchor vector can be expressed as $(x,z,v)$, where $x$ denotes the horizontal offset distance between the predicted lane and the ground-truth lane, $z$ denotes the height, and $v$ denotes the visibility of every lane point.

### Att-Gen-LaneNet network architecture

**Figure 6:**
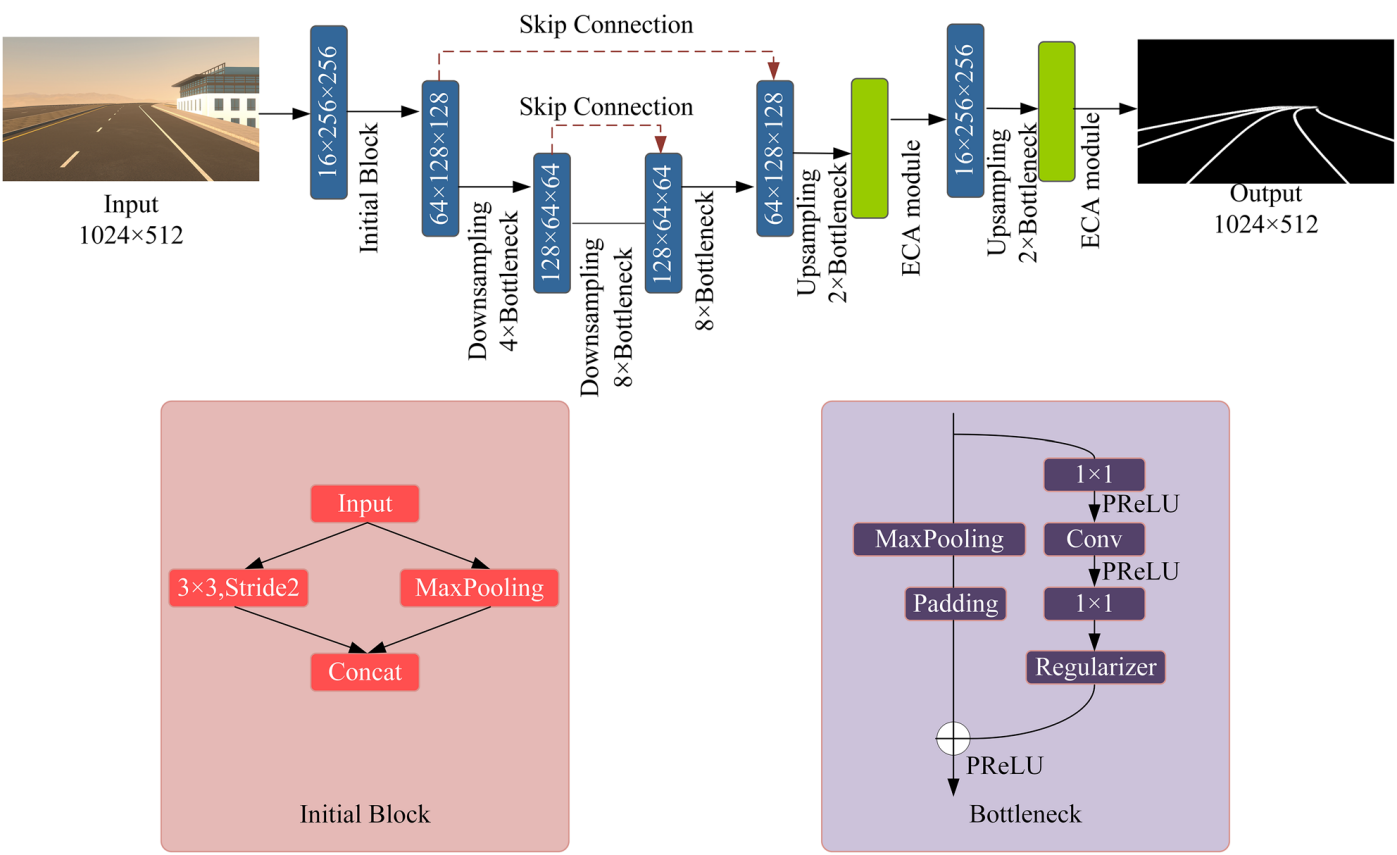
The architecture and composition of the improved ENet framework.

**Figure 7:**
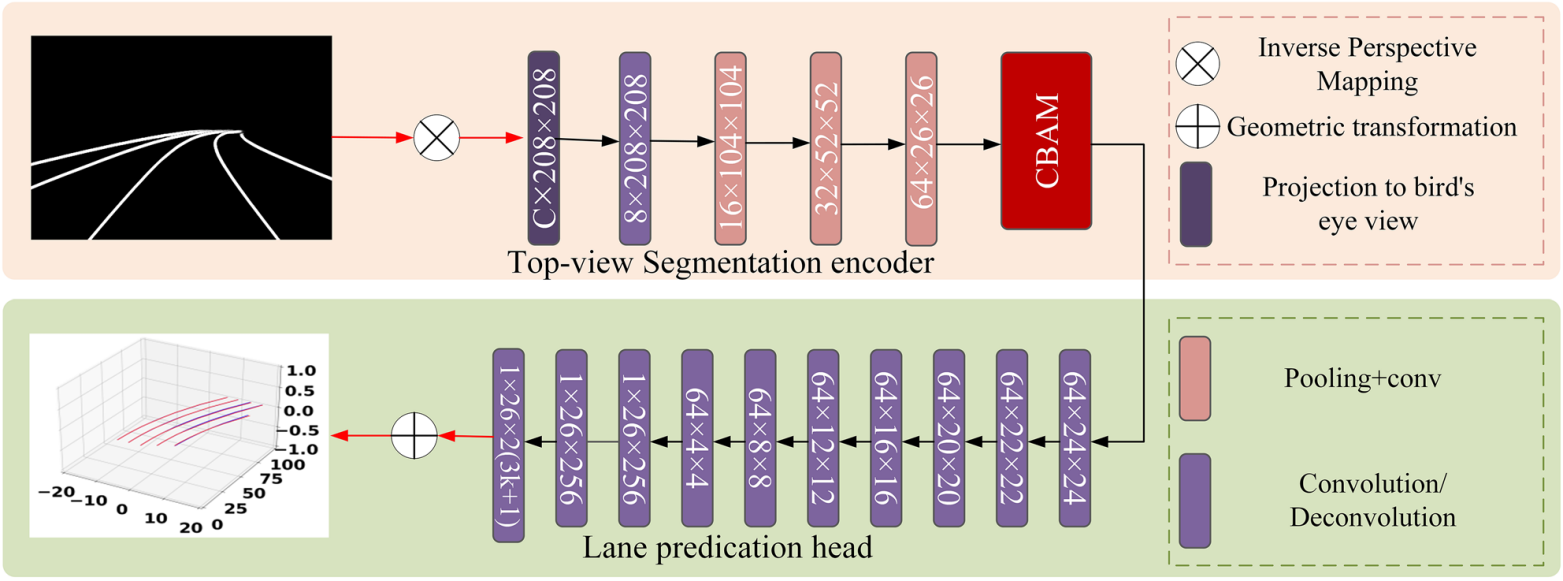
The architecture and composition of the Geometric encoding subnetwork.

In this section, Figs. 6 and 7 show the two subnetwork architectures of Att-Gen-LaneNet, and the two stages of the network need to be trained separately. The first subnetwork focuses on lane image segmentation. The second subnetwork focuses on predicting the 3D lane structure from the segmentation outputs of the first subnetwork.

We choose the improved ENet as the first subnetwork for semantic segmentation of images. The asymmetric ENet network contains a large encoder and a small decoder. The whole network consists of 6 blocks. Block1 is the initial block for generating feature maps and fusing the feature maps generated by pooling and convolution operations. Block2 and block3 are downsampling blocks, and block4 repeats the structure of block3 to increase the depth of the network. Block5 and block6 are upsampling blocks and blocks 2–6 all have bottleneck as the base structure. We use skip connection to lead the shallow features to the deeper layers of the network so that the decoder has more detailed information to obtain better segmentation and accelerate the model training. We apply the ECA attention module in the decoder to strengthen the network's ability to focus on the information of relevant channels. Figure 6 shows the first subnetwork architecture.

In the second subnetwork, the segmentation results are input to the top-view segmentation encoder and projected to the bird's eye view through inverse perspective mapping. The segmentation results are encoded in the feature maps through a series of convolution operations. The lane prediction head will use the anchor representation to predict the properties of the 3D lanes and calculate the real coordinates of the 3D lanes based on the geometric transformation. The architecture of the geometric encoding subnetwork is shown in Fig. 7.

### Loss functions of Att-Gen-LaneNet

In the first subnetwork, we use the standard cross-entropy loss function. To solve the problem of unbalanced sample distribution where the lane pixels are much less than the background pixels, we weight the loss. The equation is as follows:

5 $$W_{class} = \frac{1}{{\ln (c + p_{class} )}}$$

The weights are bounded when the probability of the lane class is close to $0$. $c$ is an additional hyperparameter, which we set to 1.06. It makes the class weights restricted to the interval [1,50].

In the second subnetwork, the proposed loss function consists of the cross-entropy loss function, geometric distance error in $x$ and $z$ directions. The cross-entropy loss function is used to evaluate the predicted lanes presence probability $p$ and visibility $v$ correctness. The following equation can express the cross-entropy loss function:

6 $$\begin{aligned} L_{cls} & = - \Sigma_{i = 1}^{N} \left( {\hat{p}^{i} \log p^{i} + \hat{p}^{^{\prime}i} \log p^{^{\prime}i} } \right) \hfill \\ &\quad - \Sigma_{i = 1}^{N} \hat{p}^{i} \cdot \left\| {\hat{v}^{i} \log v^{i} + \hat{v}^{^{\prime}i} \log v^{^{\prime}i} } \right\|_{1} \hfill \\ \end{aligned}$$

The formula $p^{^{\prime}i} = 1 - p^{i}$, and $v^{^{\prime}i} = 1 - v^{i}$.

In previous studies, researchers only used the two ends of the fitted lane and the ground truth lane for error estimation, which resulted in a large amount of valuable ground truth information being ignored. To solve this problem, we insert more points in the x-direction to reflect the quality of the fit for the whole lane. Sparse sampling and dense sampling on each anchor are denoted as $\{ y_{1}^{j} ,y_{2}^{j} , \ldots ,y_{m - 1}^{j} ,y_{m}^{j} \}$ and $\{ y_{1}^{j} ,y_{2}^{j} , \ldots ,y_{M - 1}^{j} ,y_{M}^{j} \}$, respectively. Where $M = km$, and $m$ can be chosen a suitable integer. $X_{pred}^{j}$ is the original output anchor vector of the network, and $X_{{{\text{int}} er}}^{j}$ is the anchor vector obtained by interpolation of $X_{pred}^{j}$. The ground truth anchor values $\hat{X}_{gt}^{j}$ and $\hat{X}_{gt(dense)}^{j}$ sampling at different intervals can be computed from raw ground truth 3D lane curves acquired from the synthetic environment. After interpolating the predicted 3D lanes and comparing them with the $\widehat{{\overline{x} }}$ and $\widehat{z}$ values of the ground truth 3D lanes. The following equations can express the interpolation loss function:

7 $$X_{{{\text{int}} er}}^{j} = f(X_{pred}^{j} )$$

8 $$L_{reg} = \Sigma_{j = 1}^{N} \hat{p}^{j} \cdot\left[ {\begin{array}{*{20}l} {\widehat{v}_{{\text{dense }}}^{jT} } \hfill & {\widehat{v}_{{\text{dense }}}^{jT} } \hfill \\ \end{array} } \right]\left( {\left[ {\begin{array}{*{20}l} {\overline{x}_{{\text{inter }}}^{j} } \hfill \\ {z_{{\text{inter }}}^{j} } \hfill \\ \end{array} } \right] - \left[ {\begin{array}{*{20}l} {\widehat{{\overline{x} }}_{{\text{dense }}}^{j} } \hfill \\ {\widehat{z}_{{\text{dense }}}^{j} } \hfill \\ \end{array} } \right]} \right)$$

where $f( \cdot )$ denotes the interpolation rule, and the parameter of $n$ is set to 0.6. The total loss function can be expressed as:

9 $$L_{total} = L_{class} + nL_{reg}$$

## Experiments

### Dataset selection

In this research, we choose the Apollo Synthetic dataset^22^ for 3D lane detection. There are 10,500 images from virtual scenes such as highways, cities, rural roads, and hills. These virtual scenes are created using the Unity 3D engine. The most significant advantage of the Apollo Synthetic dataset is that it provides ground truth data, including semantic/instance-level segmentation, depth and 3D lane data. The farthest distance labeled in the 3D lane label is 200 m ahead of the vehicle, the camera height is randomly set to $1.4 - 1.8\,\,{\text{m}}$, and the pitch angle is set to $0^{ \circ } - 10^{ \circ }$. Another benefit is more environmental variations, such as different times of the day, different weather conditions, different obstacles and different complex terrains.

#### Dataset division strategy

The dataset is divided according to the following three strategies to evaluate our model in different aspects.

1. We divide the unbiased images into the training set and test set according to the ratio of 5:1, as a way to perform a basic test of our algorithm.
2. The above ratio is still used to divide the number of images in the training and test sets. The training set uses unbiased data, but the test set is chosen from the traffic scene images that do not appear in the training set. We use this approach to verify the generalization ability of our method when encountering unseen scenes.
3. Many images of the same scene in the Apollo Synthetic dataset are taken at different times of the day. We store images of the same scene taken at different times of the day into the training set and test set to verify the generalization ability of our method when the scene changes visually.

### Evaluation method

We use Pixel Accuracy (PA) and MIoU as the main evaluation metrics when training the first subnetwork. Pixel Accuracy is used to calculate the ratio of the number of correctly predicted lane category pixels to the total number of pixels. For explanation, we count the lane categories as $\beta$. $P_{nm}$ indicates the number of pixels that belong to class $n$ but are mistakenly detected as class $m$, $P_{mn}$ denotes the number of pixels that belong to class $m$ but are mistakenly detected as class $n$, and $P_{nn}$ denotes the true number. MIoU is the result of the model first finding the ratio of the intersection and concatenation of the predicted and real values for each category, then summing and averaging them. $\beta { + 1}$ denotes the sum of the number of lane categories and background categories. The following equations can express Pixel Accuracy and MIoU:

10 $$PA = \frac{{\sum\nolimits_{n = 0}^{\beta } {P_{nn} } }}{{\sum\nolimits_{n = 0}^{\beta } {\sum\nolimits_{m = 0}^{\beta } {P_{nm} } } }}$$

11 $$MIoU = \frac{1}{\beta + 1}\sum\limits_{n = 0}^{\beta } {\frac{{P_{nn} }}{{\sum\nolimits_{m = 0}^{\beta } {P_{nm} + \sum\nolimits_{m = 0}^{\beta } {P_{mn} - P_{nm} } } }}}$$

In the second subnetwork, we use Average Precision (AP) and F-score as the primary metrics to evaluate the prediction results. Precision is the percentage of matched predicted 3D lanes, and recall is the percentage of matched ground truth 3D lanes. The following equation can express Average Precision:

12 $$AP = \sum\limits_{1}^{N} {p(k)} \Delta r(k)$$

where $N$ indicates the number of all images in the test set, $p(k)$ indicates the precision value when $k$ photos can be recognized and $\Delta r(k)$ indicates the change in recall value when the number of recognized images changed from $k - 1$ to $k$.

The following equation can express F-score:

13 $$F{ - }score = (1 + \alpha^{2} ) * \frac{precision*recall}{{(\alpha^{2} *precision) + recall}}$$

We adjust the value of $\alpha$ by weighing the two metrics, precision and recall. If we think that precision is more critical than recall, we adjust the value of $\alpha$ to be less than 1. If we think that recall is more critical than precision, we adjust the value of $\alpha$ to be greater than 1.

In addition, we use 40 m as the benchmark, define 40–100 m as the far distance and define 0–40 m as the near distance. We use the anchor representation to calculate the lane fitting error in the $x$ and $z$ directions of the far distance and near distance. At the predefined $y$ positions with $X^{j} = \{ x_{i}^{j} ,z_{i}^{j} ,v_{i}^{j} \}_{i = 1}^{n}$ and $X^{n} = \{ x_{i}^{n} ,z_{i}^{n} ,v_{i}^{n} \}_{i = 1}^{n}$, the error distance between $X^{j}$ and $X^{n}$ is expressed as:

14 $$dis_{jn} = \sqrt {\sum\nolimits_{i}^{n} {d_{i}^{jn} } }$$

15 $$d_{i}^{jn} = \left\{ {\begin{array}{*{20}l} {(X_{i}^{j} - X_{i}^{n} )^{2} + (Z_{i}^{j} - Z_{i}^{n} )^{2} \, ,} & {V_{i}^{j} = 1{\text{ and }}V_{i}^{n} = 1} \\ {0 \, ,} & {V_{i}^{j} = 0{\text{ and }}V_{i}^{n} = 0} \\ {d_{\max } \, ,} & {{\text{Others}}.} \\ \end{array} } \right.$$

where $v_{i}$ indicates whether the current lane reach the predefined $y$ position.

### Parameters setting

Our improved approach was compared to the three primary current baselines, where Gen-LaneNet was the primary baseline. We trained the improved ENet network and geometric encoding subnetwork separately using the Adam optimizer. The learning rates of the first and second sub-networks were set to 0.001 and 0.0005, respectively. The model was trained and tested on an NVIDIA Quadro RTX 6000 GPU. We implemented our method with Python (Version 3.7, URL https://www.python.org/downloads/release/python-3713/) and PyTorch (Version 1.4.0, URL https://download.pytorch.org/libtorch/cu101/libtorch-shared-with-deps-1.4.0.zip). The first and second sub-networks were trained for 100 and 50 epochs, respectively. The whole training process took about 9 h.

### Ablation study

**Figure 8:**
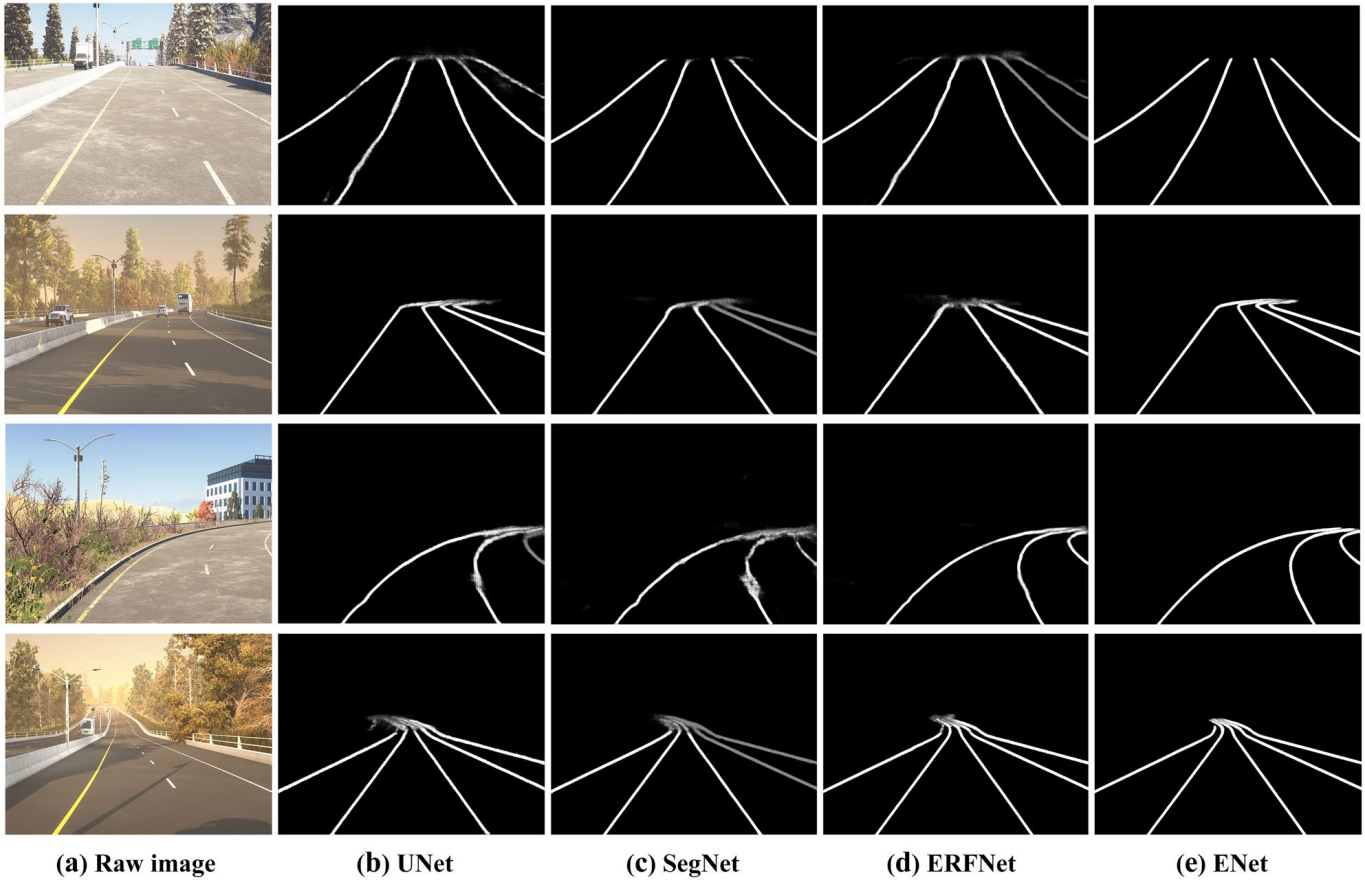
Visualization of lane semantic segmentation for complex traffic scenes. The modeling results of (**b**) UNet, (**c**) SegNet, (**d**) ERFNet and (**e**) ENet performed by Python 3.7 and PyTorch 1.4.0.

Due to excessive ablation experiments, we only show the segmentation results of UNet^27^, SegNet^28^, ERFNet^29^, and ENet^23^ after adding the ECA attention module and the weighted cross-entropy loss function. The results in Fig. 8 show that the improved ENet’s performance well in complex traffic scenes and solves the problem of ambiguous distant lane segmentation very well.

We choose UNet, SegNet, ERFNet, and ENet to conduct many ablation experiments. In Table 1, "ECA" indicates the addition of the ECA attention module, "ECA/Wclass" indicates the addition of the ECA attention module and the weighted cross-entropy loss function. We verify the performance improvement of each part by the above method.

**Table 1:** Comparison of different improvements in the course of ablation experiments.

Method	PA	MIoU	Epochs
UNet	97.6	71.7	50
UNet(ECA)	97.8	72.2	50
UNet(ECA/Wclass)	98.4	74.6	50
SegNet	98.2	78.9	50
SegNet(ECA)	98.8	82.2	50
SegNet(ECA/Wclass)	99	85.1	50
ERFNet	97.6	70.4	50
ERFNet(ECA)	98.8	81.4	50
ERFNet(ECA/Wclass)	98.4	80.4	50
ENet	98.8	84.1	100
ENet(ECA)	99.2	87.9	100
ENet(ECA/Wclass)	**99.7**	**89.5**	100

Significant values are in bold.

#### Benefits of the ECA attention module

When adding only the ECA attention module to the original networks, the Pixel Accuracy can be improved by 0.6% on average, and the MIoU can be improved by 4.6% on average.

#### Benefits of the weighted cross-entropy loss function

When adding the ECA attention module and the weighted cross-entropy loss function to the original networks, the Pixel Accuracy can be improved by 0.8% on average, and the MIoU can be improved by 6.1% on average.

**Figure 9:**
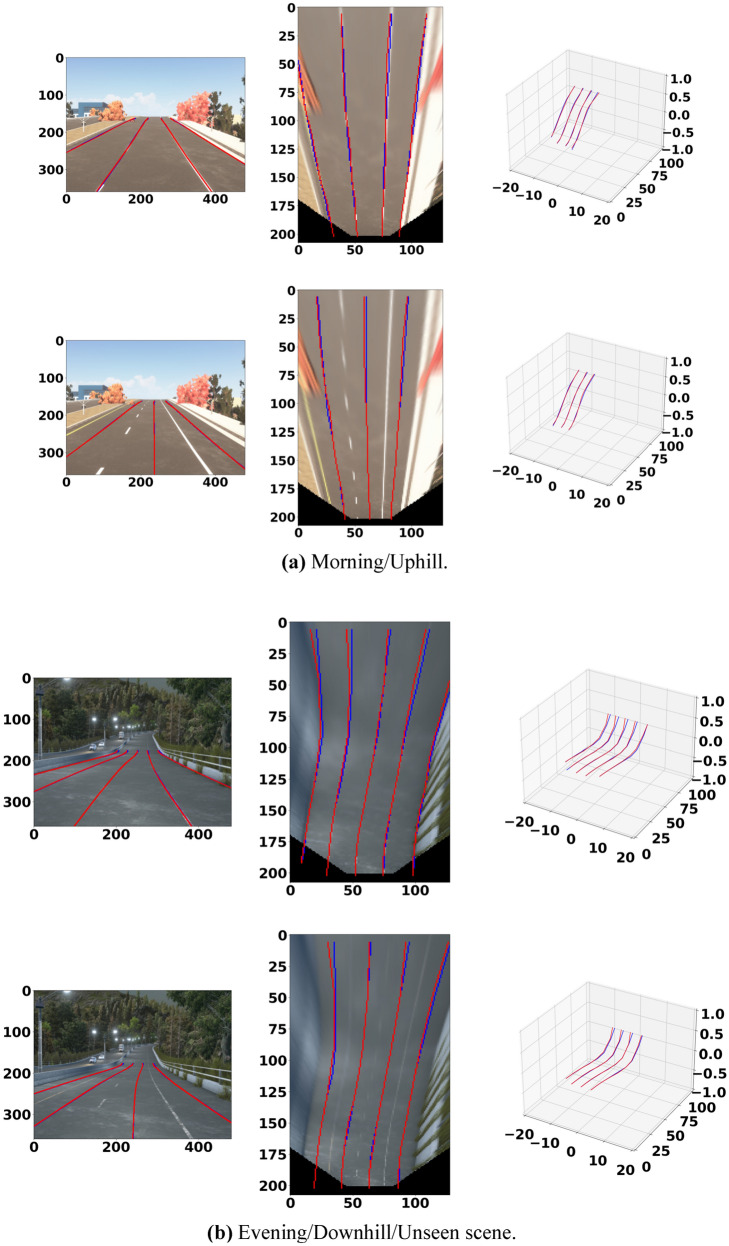

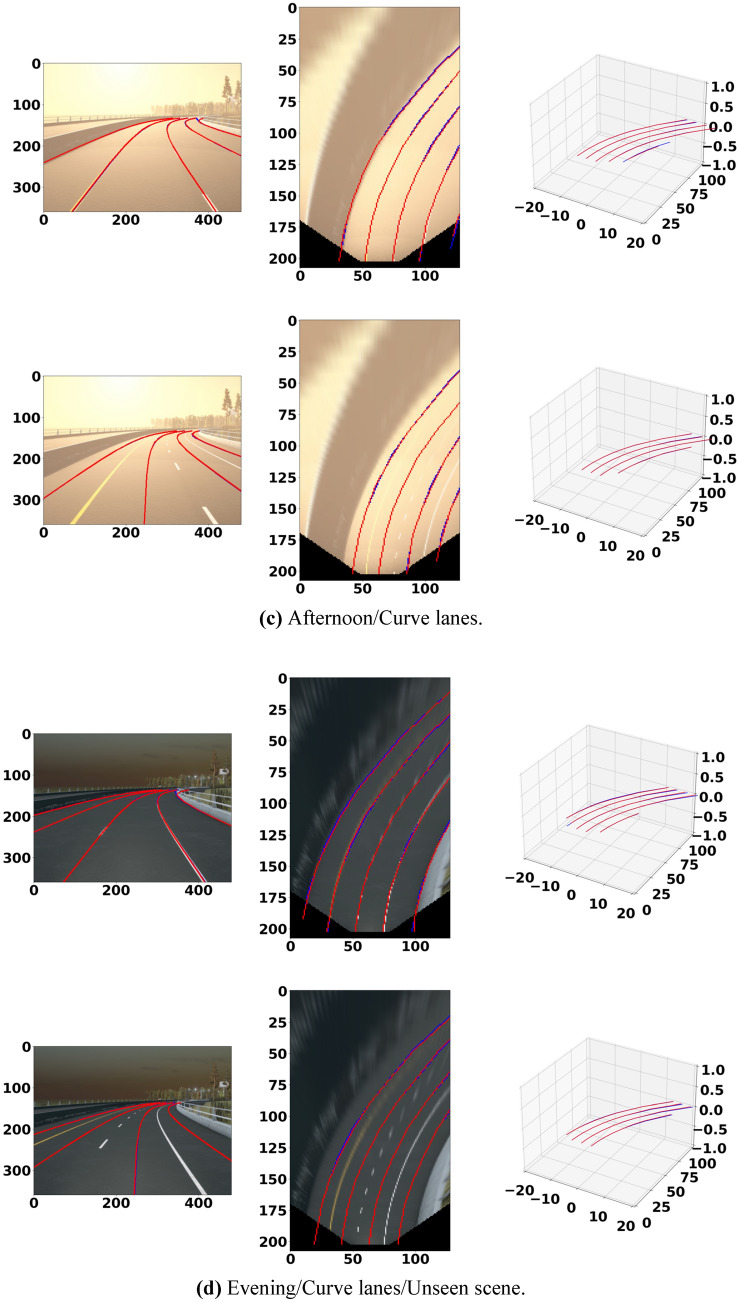
Examples of 3D lane detection prediction. The modeling results of Att-Gen-LaneNet performed by Python 3.7 and PyTorch 1.4.0.

The 3D lane prediction results in Fig. 9a,b show that our model has superior generalization ability in complex traffic scenes such as uphills and downhills, unseen scenes, and visual changes. Figure 9c,d show that our model has superior generalization ability in complex traffic scenes such as curve lanes, unseen scenes, and visual changes.

In Table 2, "CA" indicates the addition of the CBAM attention module, and "CA/IL" indicates the addition of the CBAM attention module and the interpolation loss function. The increasing trend of the data in Table 2 fully illustrates that our improvements have improved the model performance in all scenes.

**Table 2:** Comparison of the generalization performance of three dataset division strategies.

		F-score	AP	X error (near)	X error (far)	Z error (near)	Z error (far)
Balanced scenes	3D-LaneNet	85.8	89.1	0.071	0.481	0.014	0.207
	3D-LaneNet(CA)	89.2	91.1	0.088	0.503	0.014	0.199
	3D-LaneNet(CA/IL)	90.6	92.4	0.085	0.439	0.013	0.168
	Gen-LaneNet	88.4	90.3	0.062	0.498	0.014	0.252
	Gen-LaneNet(CA)	89.5	91.7	0.083	0.477	0.014	0.217
	Gen-LaneNet(CA/IL)	91.2	93.2	0.074	0.441	0.013	0.198
	Att-Gen-LaneNet	**92.4**	**94.1**	0.053	0.412	0.013	0.232
Unseen scenes	3D-LaneNet	72.2	74.4	0.168	0.857	0.037	0.523
	3D-LaneNet(CA)	82.2	83.9	0.241	0.917	0.034	0.547
	3D-LaneNet(CA/IL)	84.7	86.6	0.216	0.888	0.028	0.51
	Gen-LaneNet	78.4	79.1	0.141	0.905	0.028	0.535
	Gen-LaneNet(CA)	81.3	83.3	0.24	0.857	0.024	0.611
	Gen-LaneNet(CA/IL)	81.8	84.2	0.274	0.844	0.028	0.575
	Att-Gen-LaneNet	**86.5**	**87.8**	0.123	0.853	0.026	0.62
Visual variations	3D-LaneNet	72.4	74.7	0.117	0.598	0.033	0.234
	3D-LaneNet(CA)	80.4	83.1	0.107	0.588	0.029	0.284
	3D-LaneNet(CA/IL)	82.1	84.6	0.099	0.592	0.019	0.227
	Gen-LaneNet	85.2	87.3	0.072	0.541	0.015	0.303
	Gen-LaneNet(CA)	90	92.2	0.077	0.509	0.024	0.29
	Gen-LaneNet(CA/IL)	90.2	93.1	0.084	0.447	0.017	0.227
	Att-Gen-LaneNet	**90.6**	**92.6**	0.06	0.441	0.012	0.251

Significant values are in bold.

#### Benefits of the CBAM attention module

When adding only the CBAM attention module to 3D-LaneNet and Gen-LaneNet, the F-score can achieve an average of 5% improvement in the three scenes, especially in the Unseen scenes, 3D-LaneNet achieves 10.2% improvement. AP achieves an average of 5% improvement in the three scenes, especially in the Visual variations, and 3D-LaneNet achieves 8.2% improvement.

#### Benefits of the interpolation loss function

When adding the CBAM attention module and the interpolation loss function to 3D-LaneNet and Gen-LaneNet, F-score achieves an average of 1.3% improvement in the three scenes compared to adding the CBAM attention module only. AP achieves an average of 1.5% improvement in the three scenes.

Most importantly, our improved Att-Gen-LaneNet shows the best results in the three scenes.

## Conclusions

In this paper, the proposed two-stage network Att-Gen-LaneNet perfectly solved the problem of ambiguous distant lane segmentation and achieved robust 3D lane structure prediction in complex traffic scenes. The model had strong generalizability and could be extended to unseen scenes. This work is beneficial to the combination and development of deep learning and autonomous driving technologies. The method proposed in this paper achieves excellent results, but it is very demanding on data. This method not only requires a large number of 3D scene images but also these images need to provide accurate annotation information, so the weakly supervised-based method will be a development direction.

## Data Availability

All data included in this study are available upon request by contact with the corresponding author.
